# The Effects of Heat Treatment on the Physical Properties and Surface Roughness of Turkish Hazel (*Corylus colurna* L.) Wood

**DOI:** 10.3390/ijms9091772

**Published:** 2008-09-16

**Authors:** Derya Sevim Korkut, Süleyman Korkut, Ilter Bekar, Mehmet Budakçi, Tuncer Dilik, Nevzat Çakicier

**Affiliations:** 1 Faculty of Forestry, Department of Forest Products Engineering, Duzce University, 81620, Duzce, Turkey. E-Mails: deryasevimkorkut@duzce.edu.tr (D. K.); ilterbekar@duzce.edu.tr (I. B.); nevzatcakicier@duzce.edu.tr (N. C.); 2 Faculty of Technical Education, Department of Furniture and Design, Duzce University, 81620, Duzce, Turkey. E-Mail: mbudakci@gazi.edu.tr; 3 Faculty of Forestry, Department of Forest Products Engineering, Istanbul University, 34473, Bahcekoy, Sariyer, Turkey. E-Mail: tuncerd@istanbul.edu.tr

**Keywords:** Turkish Hazel, *Corylus colurna* L., heat treatment, physical properties, surface roughness

## Abstract

Heat treatment is often used to improve the dimensional stability of wood. In this study, the effects of heat treatment on the physical properties and surface roughness of Turkish Hazel (*Corylus colurna* L.) wood were examined. Samples obtained from Kastamonu Forest Enterprises, Turkey, were subjected to heat treatment at varying temperatures and for different durations. The physical properties of heat-treated and control samples were tested, and oven-dry density, air-dry density, and swelling properties were determined. A stylus method was employed to evaluate the surface characteristics of the samples. Roughness measurements, using the stylus method, were made in the direction perpendicular to the fiber. Four main roughness parameters, mean arithmetic deviation of profile (Ra), mean peak-to-valley height (Rz), root mean square roughness (Rq), and maximum roughness (Ry) obtained from the surface of wood were used to evaluate the effect of heat treatment on the surface characteristics of the specimens. Significant difference was determined (p = 0.05) between physical properties and surface roughness parameters (Ra,Rz, Ry, Rq) for three temperatures and three durations of heat treatment. The results showed that the values of density, swelling and surface roughness decreased with increasing temperature treatment and treatment times. Turkish Hazel wood could be utilized successfully by applying proper heat treatment techniques without any losses in investigated parameters. This is vital in areas, such as window frames, where working stability and surface smoothness are important factors.

## 1. Introduction

Hazelnut is a name given to the genus *Corylus* (Betulaceae) which includes about ten species. The Turkish hazel (*Corylus colurna* L.) tree is one of the wild species within the genus *Corylus*. Turkish hazel (*Corylus colurna* L.) is native to southeast Europe and southwest Asia, from the Balkans through northern Turkey to northern Iran. It is the largest species of hazel, reaching a height of 35 m, with a stout trunk of up to 1.5 m diameter. It prefers well drained chalky soils. Stems and roots are used by furniture makers for decorative inlays and veneers. The fine lustrous wood is pinkish brown and it polishes beautifully [1].

Heat treatment of wood is an effective method to improve the dimensional stability and durability against biodegradation. Considerable research has focused on the application of heat treatments to improve the dimensional stability, hygroscopic properties and biological resistance of wood. Heat treatments of wood modify chemistry of cell components. These chemical changes can be expressed as increased dimensional stability and decreased hygroscopicity [2–4].

Within the last several years five different types of heat treatments have gained industrial significance in Europe. Some of the products developed by thermal treatment include thermowood in Finland, retification process and bois perdure in France, oil-heat treatment in Germany and plato-wood in The Netherlands [5–7].

The temperature and duration for heat treatment generally vary from 180 to 280 °C and 15 min to 24 h depending on the heat treatment process, wood species, sample size, moisture content of the sample, and the desired mechanical properties, resistance to biological attack, and dimensional stability of the final product [8]. Temperature has a greater influence than time on many properties. Treatment at lower temperatures for longer periods, however, does not give similar results compared to treatments at higher temperatures.

Wood treated at high temperature has less hygroscopicity than natural wood. Heating wood permanently changes several of its chemical and physical properties. The change in properties is mainly caused by thermic degradation of hemicelluloses. Theoretically, the available OH groups in hemicellulose have the most significant effect on the physical properties of wood. Heat treatment lowers water uptake and wood cell wall absorbs less water because of the decrease of the amount of wood’s hydroxyl groups. As a consequence of the reduced number of hydroxyl groups the swelling and shrinking are lower. In addition to better durability the advantages of heat treated wood are reduced hygroscopicity and improved dimensional stability. It stabilizes around 4–5% in humidity instead of 10–12% [9].

Heat-treated wood ‘lives’ less than untreated wood, meaning that shrinking and swelling is considerably lower. Stamm and Hansen [10] reported that the hygroscopicity of black gum wood decreased to half of its original value when samples were treated at 205 °C for 6 h. In another study increases in temperature and treatment time, and also the technique used, resulted in changes in dimensional stability from 55% to 90% [11]. When young beech, oak, and pine wood was heat treated at 70 to 200 °C between 6 and 48 h, the effect of heat treatment was obvious after 100 °C, and sorption capacity decreased with increased treatment time and temperature [12].

In addition, heat treatment resulted in varying amounts of weight loss, depending on the treatment temperature and time. In a study on spruce (*Picea abies*) wood, heat treatment for 24 h resulted in a weight loss of 0.8% and 15.5% at 120 °C and 200 °C, respectively [13]. Weight loss of beech (*Fagus sylvatica*) wood, treated at increasing temperatures, was 8.1% and 9.8% at 150 °C and 200 °C, respectively [14, 15] reported mass losses of 6.4, 7.1 and 10.2% for *Betula pendula* treated at 205 °C for 4, 6 and 8 h.

Heat-treated wood is a new, ecological wood product, which main advantage is the ecological method of manufacture. Heat-treated wood is generally used indoors on parquet and wooden floors, wall and ceiling panels, in saunas and fixed installations for example in the kitchen. It can also be used to make furniture and other utensils, decorations and giftware. Heat-treated wood is rot-resistant enough for many outdoor uses, and this has been achieved without using chemicals that harm the environment or people’s health. Heat-treated wood is also an ecological alternative to tropical woods because of the beautiful and pleasant colour it gives [16].

To our knowledge, there is no information about the influence of heat treatment on some physical properties, such as density, swelling, and surface roughness of Turkish hazel wood. This study will hopefully offer the timber products industry many interesting opportunities after demonstrating that heat-treated Turkish hazel woods have improved characteristics.

## 2. Experimental Section

Five trees with a diameter at breast height diameter (DBH. 1.3 m above ground) of 30–35 cm were obtained from Kastamonu Forest Enterprises [17]. The area from which the trees were taken was at an elevation of 1,290 m and had a slope of 30%. Lumber from the logs was prepared by Oney Kaplama San. A. Ş. Turkish Hazel lumber was finished by a fixed-knife planer with a feed speed of 1 m/s. The bias angle of the knife was 45° for the lumber. If the wood pieces are sawn so that the annual rings are at least in 45° angle to the surface the deformations will be smaller, the hardness of the surface will be stronger and the “general looks” after heat treatment is better. Sampling and tests were performed according to several Turkish and ISO standards. Small clear samples were obtained for density and swelling (20×20×30 mm), and surface roughness measurements (50×50×50 mm).

The samples were subjected to heat treatment at 120 °C, 150 °C, or 180 °C for 2, 6, or 10 h in a small heating unit controlled to within ±1 °C under atmospheric pressure. After heat treatment, treated and untreated samples were conditioned at 20 ± 2 °C and 65% (±5%) relative humidity (RH) in a conditioning room to reach equilibrium moisture content (EMC) of 12%. The air-dry density of the samples was determined. The dimensions and weights of the samples were measured. The oven-dry and air-dry density of the samples was determined at 0.01 mm and 0.001 g sensitivity. After the oven-dry dimensions were determined, the samples were soaked in water (20 ± 2 °C). When no changes in sample dimensions were observed, the dimensions were measured. Tests for density (30 samples) and swelling (30 samples) were carried out based on ISO 3131 [18] and TS 4084 [19], respectively.

Surface roughness of the samples was measured using a profilometer (Mitutoyo Surftest SJ-301). Measurements were made with the profile method using a stylus device standard. The measuring speed, pin diameter, and pin top angle of the tool were 10 mm/min, 4 μm, and 90°, respectively. The points of roughness measurement were randomly marked on the surface of the samples. Measurements were carried out in the direction perpendicular to the fiber of the samples.

Three roughness parameters, mean arithmetic deviation of profile (Ra), mean peak-to-valley height (Rz), and maximum roughness (Ry) were commonly used in previous studies to evaluate surface characteristics of wood and wood composites including veneer [20]. Ra is the average distance from the profile to the mean line over the length of assessment. Rq is the square root of the arithmetic mean of the squares of profile deviations from the mean line. Rz can be calculated from the peak-to-valley values of five equal lengths within the profile while maximum roughness (Ry) is the distance between peak and valley points of the profile which can be used as an indicator of the maximum defect height within the assessed profile [21]. Therefore, such parameters which are characterized by ISO 4287 [22] and DIN 4768 [23] were recorded.

**Figure 1.**
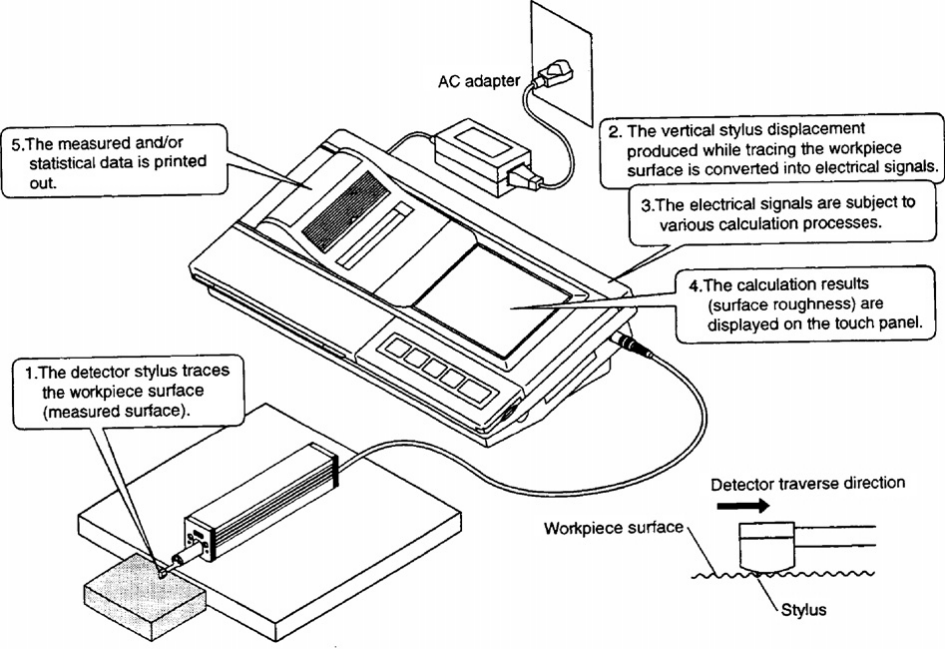
Schematic description of the Mitutoyo Surftest SJ-301.

Specification of this parameter is described by Hiziroglu [24], and Hiziroglu and Graham [25]. Roughness values were measured with a sensitivity of 0.5 lm. The length of scanning line (Lt) was 15 mm and the cut off was k = 2.5 mm. The measuring force of the scanning arm on the surfaces was 4 mN (0.4 g), which did not put any significant damage on the surface according to Mitutoyo Surftest SJ-301 user manual [26]. Measurements were performed at room temperature and the pin was calibrated before the tests. Figure 1 shows the Mitutoyo Surftest SJ-301.

For the oven-dry density, air-dry density, swelling and average roughness, all multiple comparisons were first subjected to an analysis of variance (ANOVA) and significant differences between mean values of control and treated samples were determined using Duncan’s multiple range test.

## 3. Results and Discussion

Table 1 shows the oven-dry and air-dry densities and swelling ratios under the different heat treatment and time combinations. According to the averages, all the parameters decreased with increasing temperature and time. It is evident from the results that these values were all lower in heat-treated samples than in control samples. The effect of the heat treatments was significant for all the variables analyzed. It is clear from this study that the value of all measured physical properties and surface roughness decreased with increasing temperature and duration.

Heating wood permanently changes several of its chemical and physical properties. The change in properties is mainly caused by thermic degrading of hemicelluloses. Theoretically, the available OH groups in hemicellulose have the most significant effect on the physical properties of wood. Heat treatment slows water uptake and wood cell wall absorbs less water because of the decrease of the amount of wood’s hydroxyl groups. As a consequence of the reduced number of hydroxyl groups the swelling and shrinking are lower. In addition to better durability the advantages of heat-treated wood are reduced hygroscopicity and improved dimensional stability. Heat treatment significantly reduces the tangential and radial swelling. The wood’s swelling and shrinkage is very low. Desired changes start to appear already at about 150 °C, and the changes continue as the temperature is increased in stages [27].

Table 2 shows the percentage decrease of values in relation to the control for each treatment and each measured parameter.

It is evident from Table 1 that the oven-dry density and air-dry density values decrease with increasing temperature and heat treatment time under the conditions used. Heat treated wood samples at a temperature of 180 °C for 10 h gave the lowest air-dry and oven-dry density values when compared with other conditions studied.

It is known that the weight of wood material decreases when heat treatment is applied. These changes resulting from heat treatment could be explained by losses in the cell wall, extractive substances and hemicellulose degradation due to the high temperature applied.

Decreases in swelling to radial, tangential and longitudinal directions were found to be 20.29%, 23.53%, and 55.13%, respectively, when treated at 180 °C for 10 h. A decrease in swelling results in an increase in dimensional stability, which is required for several uses of wood.

Surface roughness can be affected by various factors such as annual ring width, differences between juvenile and mature wood, density, differences between early and late wood and specific cell structures [28].

Surface roughness decreased by nearly 37% in the sample heat-treated at 180 °C for 10 h when compared with the control samples. This increase in smoothness or decrease in roughness is very important for many applications of solid wood. In addition, losses occurring in the planning machine are reduced and high quality surfaces are attained.

It may be concluded that the heat treatment resulted a plastification on the solid wood surfaces. High temperatures above 160 °C cause lignin to a thermoplastic condition and thus to densify and compact solid wood surface [29, 30].

Also, the wooden materials with rough surface requires much more sanding process compared to one with smooth surface, which leads to decrease in thickness of material and, therefore, increase the losses due to the sanding process [28, 31].

The maximum decreases for all parameters were recorded at the treatment of 180 °C for 10 h. The lowest oven-dry density values obtained was 0.511 gr/cm^3^, total loss compared to the control was calculated to be 26.90%. Similarly, the lowest air-dry density was also obtained for samples treated at 180 °C for 10 h (0.534 gr/cm^3^). The air-dry density loss was 27.36% when compared to the control.

**Figure 2.**
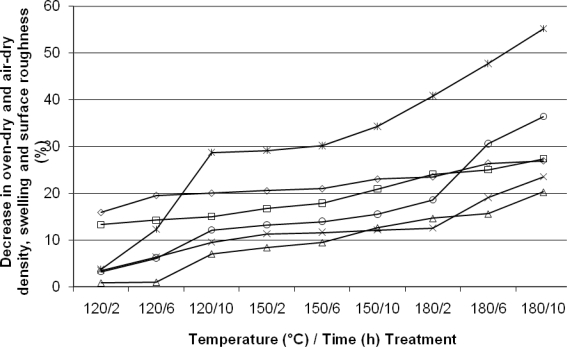
Percentage decrease of physical properties in Turkish Hazel (*Corylus colurna* L.) wood following heat treatment for different durations. (⋄) oven-dry density; (□) air-dry density; (Δ) radial swelling; (x) tangential swelling; (*) longitudinal swelling; (o) surface roughness (Ra)

The parameters measured varied in their rates of decrease with some experiencing a gradual loss and others exhibiting more dramatic changes (Figure 2). Heat treatment resulted in varying amounts of weight loss, depending on the treatment temperature and time.

In general the results of this study on the effect of heat treatment on Turkish Hazel are compatible with the findings in literature on the effect of heat treatment on different tree species. Yildiz [31] reported that the density observed for beech (2.25%) and spruce (1.73%) woods treated at 130 °C for 2 h was higher than that of control samples. On the other hand, at longer treatment times and higher temperatures, the density decreased. The highest decrease in density was observed in samples treated at 200° for 10 h (beech, 18.37% decrease; beech spruce, 10.53% decrease). The increase in dimensional stability was calculated to be 50% for beech and 40% for spruce wood. Similar results were also observed when samples were treated in an inert gas atmosphere at 180–200 °C and 8–10 bar of atmospheric pressure (beech, 10–15% density decrease; spruce, 5–10% decrease) [32]. In another study, the dimensional stability was 60% higher for oak heartwood, 55% higher for pine heart- and sapwood, and 52% higher for spruce heart- and sapwood after heat treatment [33].

Unsal and Ayrilmis [34] also found that the maximum surface roughness decrease in Turkish river red gum (*Eucalyptus camaldulensis* Dehn.) wood samples was 27.9% at 180 °C for 10 h. Korkut [35] obtained similar oven-dry and air-dry density, swelling and surface roughness values for Uludag fir (*Abies bornmuellerinana* Mattf.) wood for the same treatment time and temperature.

Unsal *et al*. [36] reported that in Turkish river red gum (*Eucalyptus camaldulensis* Dehn.) wood samples the largest swelling loss was at 180 °C after 10 h treatment. The loss was 14.11% radially, and 21.51% tangentially. Oven-dry density decreased by up to 11.76% in the sample heat-treated at 180 °C for 10 h when compared with the control samples.

Esteves *et al*. [37] reported that in *Pinus pinaster* and *Eucalyptus globulus* wood samples in the absence of air by steaming, treatment inside an autoclave heated at 190–210 °C for 2–12 h resulted in an equilibrium moisture content decrease by 46% for pine and 61% for eucalyptus, while the dimensional stability increased (maximum anti-shrinking efficiency in the radial direction of 57 and 90% for pine and eucalyptus, respectively) and the surface wettability was lowered. Mass losses increased with treatment time and temperature, reaching 7.3% for pine and 14.5% for eucalyptus wood. The wood behaviour towards moisture was improved.

These results can be explained by material losses in the cell lumen and hemicellulose degradation due to the high applied temperature. It is known that the weight of wood material and its swelling decreases when heat treatment is applied. Heat treatment lowers water uptake and wood cell wall absorbs less water because of the decrease of the amount of hydroxyl groups in the wood. As a consequence of the reduced number of hydroxyl groups, the swelling and shrinking were lower [11].

## 4. Conclusions

In conclusion, it was found that the density, swelling and surface roughness of the Turkish Hazel decreased for all treatment conditions (temperatures and times). The smallest decrease was observed in the treatment at 120 °C for 2 h. The largest decrease found was for swelling, followed by Ra and the air-dry density, when samples were heat-treated under the specific conditions of this research.

The improved characteristics in swelling and surface roughness of heat treated timber have to be balanced against the decrease in strength values when evaluating the effectiveness of using this treatment. This wood species can be utilized by applying adequate heat-treatment techniques that result in negligible losses in density values in areas where working, stability, and surface smoothness are important factors. Due to its good weather resistance, thermowood is suited for outdoor applications such as external cladding, paneling, parquets, sauna, window frames and garden furniture.

The improved characteristics of heat treated timber offer the timber product industry many potential and attractive new opportunities. The most important property, when compared to untreated wood, is that the equilibrium moisture content of the heat-treated wood is reduced and as a consequence of this shrinkage and swelling of the wood is also reduced. The best way of utilizing heat-treated timber is to make use of these improved properties.

## Figures and Tables

**Table 1.** The effect of heat treatment for different durations on physical properties and surface roughness in Turkish Hazel (*Corylus colurna* L.) wood^a^.

Heat Treat-ment	Times	Unit^b^	Oven-dry Density^c^	Air-dry Density^c^	Surface Roughness^c^				Swelling^c^	
			Do (g/cm^3^)	D_12_ (g/cm^3^)	Ra (μm)	Ry (μm)	Rz (μm)	Rq (μm)	Radial (%)	Tangential (%)	Longitudinal (%)
Control		Avg.	0.699	0.735	10.398	86.176	66.380	13.226	5.512	9.373	1.265
			A	A	A	A	A	BA	A	A	A
		± s	0.062	0.063	2.613	19.275	4.909	1.213	0.266	0.494	0.237
		s^2^	0.004	0.004	6.828	371.530	24.103	1.470	0.071	0.244	0.056
		V	8.932	8.571	25.130	22.367	7.396	9.168	4.818	5.268	18.697
		N	30	30	30	30	30	30	30	30	30
120 °C	2 hr.	Avg.	0.587	0.637	10.062	83.680	65.294	12.427	5.464	9.044	1.217
			BCDE	BFG	ABCD	AEF	ADEF	AEF	ABCD	ACDE	ACDE
			FGHIK	HIK	EFGH	GH	GHI	GH	EFGH	FGHI	FGHI
		± s	0.029	0.052	0.902	12.777	15.867	0.608	0.341	1.801	0.285
		s^2^	0.001	0.003	0.814	163.260	251.758	0.369	0.116	3.244	0.081
		V	4.961	8.202	8.965	15.269	24.301	4.889	6.240	19.915	23.382
		N	30	30	30	30	30	30	30	30	30
	6 hr.	Avg.	0.562	0.630	9.758	83.240	61.762	12.366	5.456	8.775	1.110
			CGH	CFG	AEF	AEF	BFG	AEF	ABCD	BHI	BCDE
			IK	HIK	GH	GH	HI	GH	EFGH		FGHI
		± s	0.059	0.076	1.962	7.073	4.602	2.390	0.156	0.740	0.256
		s^2^	0.003	0.006	3.848	50.028	21.177	5.712	0.024	0.547	0.066
		V	10.482	12.006	20.104	8.497	7.451	19.326	2.855	8.430	23.104
		N	30	30	30	30	30	30	30	30	30
	10 hr.	Avg.	0.559	0.624	9.140	79.500	60.982	11.830	5.122	8.481	0.902
			DHIK	DGHIK	BGH	BEFGH	CFGHI	BFGH	BEFGH	CHI	CGHI
		± s	0.024	0.048	0.889	18.919	10.582	1.139	0.595	0.967	0.146
		s^2^	0.001	0.002	0.790	357.928	111.978	1.298	0.354	0.935	0.021
		V	4.288	7.673	9.723	23.797	17.353	9.632	11.622	11.398	16.173
		N	30	30	30	30	30	30	30	30	30
150 °C	2 hr.	Avg.	0.555	0.612	9.026	79.288	60.496	11.782	5.046	8.308	0.897
			EIK	EGHIK	CGH	CEFGH	DFGHI	CFGH	CEFGH	DHI	DGHI
		± s	0.031	0.014	1.689	10.358	9.119	1.088	0.173	0.744	0.179
		s^2^	0.001	0.000**2**	2.853	107.294	83.149	1.183	0.030	0.554	0.032
		V	5.666	2.296	18.713	13.064	15.073	9.232	3.422	8.958	19.907
		N	30	30	30	30	30	30	30	30	30
	6 hr.	Avg.	0.552	0.603	8.946	79.136	60.252	11.658	4.988	8.282	0.884
			FIK	FHIK	DGH	DEFGH	EFGHI	DFGH	DFGH	EHI	EHI
		± s	0.042	0.057	0.794	10.910	6.771	2.020	0.435	1.044	0.258
		s^2^	0.002	0.003	0.630	119.034	45.843	4.079	0.189	1.091	0.066
		V	7.543	9.438	8.870	13.787	11.237	17.324	8.722	12.611	29.153
		N	30	30	30	30	30	30	30	30	30
	10 hr.	Avg.	0.538	0.581	8.786	70.820	54.694	11.094	4.813	8.233	0.832
			GIK	GIK	EGH	EFGH	FHI	EGH	EH	FHI	FHI
		± s	0.025	0.042	1.798	9.447	8.197	1.800	0.217	1.666	0.321
		s^2^	0.001	0.002	3.234	89.242	67.186	3.239	0.047	2.776	0.177
		V	4.629	7.199	20.470	13.339	14.986	16.223	4.512	20.239	38.581
		N	30	30	30	30	30	30	30	30	30
180 °C	2 hr.	Avg.	0.534	0.558	8.468	63.938	54.234	10.714	4.703	8.195	0.749
			HIK	H	FGH	FH	GHI	FGH	FH	GHI	GI
		± s	0.037	0.027	2.405	8.365	3.999	2.743	0.462	0.820	0.227
		s^2^	0.001	0.001	5.785	69.967	15.993	7.525	0.213	0.672	0.107
		V	6.994	4.765	28.403	13.082	7.374	25.603	9.822	10.004	30.307
		N	30	30	30	30	30	30	30	30	30
	6 hr.	Avg.	0.514	0.551	7.216	59.146	48.235	9.168	4.650	7.582	0.662
			I	I	G	G	H	G	GH	H	H
		± s	0.036	0.039	0.787	6.567	6.616	0.902	0.625	0.581	0.250
		s^2^	0.001	0.001	0.619	43.128	43.765	0.813	0.390	0.338	0.062
		V	6.964	7.008	10.902	11.103	13.715	9.837	13.436	7.666	37.718
		N	30	30	30	30	30	30	30	30	30
	10 hr.	Avg.	0.511	0.534	6.615	56.846	47.106	8.724	4.393	7.168	0.568
			K	K	H	H	I	H	H	I	I
		± s	0.019	0.010	0.900	7.848	3.111	1.240	0.138	0.510	0.147
		s^2^	0.0004	0.0001	0.810	61.595	9.678	1.537	0.019	0.260	0.021
		V	3.789	1.905	13.605	13.806	6.604	14.211	3.146	7.116	25.809
		N	30	30	30	30	30	30	30	30	30

a Number of samples used in each test is 20.

b Avg. = average; ±s = standard deviation; s^2^=variance. V= coefficient of variation. N= number of samples used in each test.

c Homogenous groups: letters in each column indicate groups that are statistically different according to Duncan’s multiple range test at P < 0.05. Comparisons were between each control and its test.

**Table 2.** Percentage decrease of physical properties and surface roughness in Turkish Hazel (*Corylus colurna* L.) wood following heat treatment for different durations.

Heat Treat-ment	Time (h)	Oven-dry density	Air-dry density	Swelling			Surface Roughness			
				Radial	Tangential	Longitudinal	Ra	Ry	Rz	Rq
		%	%	%	%	%	%	%	%	%
120 °C	2	15.95	13.33	0.87	3.51	3.80	3.23	2.90	1.64	6.04
	6	19.57	14.28	1.02	6.38	12.30	6.16	3.41	6.96	6.50
	10	20.04	15.04	7.08	9.51	28.68	12.10	7.75	8.13	10.55
150 °C	2	20.54	16.72	8.44	11.36	29.12	13.19	7.99	8.86	10.92
	6	20.99	17.86	9.49	11.64	30.16	13.96	8.17	9.23	11.86
	10	23.02	20.94	12.67	12.17	34.26	15.50	17.82	17.60	16.12
180 °C	2	23.51	24.09	14.67	12.56	40.82	18.56	25.81	18.30	18.99
	6	26.41	25.06	15.63	19.11	47.71	30.60	31.37	27.34	30.68
	10	**26.90**	**27.36**	**20.29**	**23.53**	**55.13**	**36.38**	**34.03**	**29.04**	**34.04**
